# Hepatitis B Prevalence and Referral Rates in Vulnerable Populations Undergoing Community-Based Screening—Results from the LIVE(RO)2 Program

**DOI:** 10.3390/v16081318

**Published:** 2024-08-18

**Authors:** Speranta Iacob, Irma Csiki, Razvan Iacob, Mihaela Ghioca, Ileana Constantinescu, Bogdan Chiper, Laura Huiban, Cristina Muzica, Irina Girleanu, Nicoleta Tiuca, Sorina Diaconu, Larisa Sandulescu, Ion Rogoveanu, Florentina Furtunescu, Corina Pop, Anca Trifan, Liana Gheorghe

**Affiliations:** 1 Fundeni Clinical Institute, 022328 Bucharest, Romania; iecsiki@gmail.com (I.C.); raziacob@gmail.com (R.I.); lita.mihaela.corina@gmail.com (M.G.); ileana.constantinescu@imunogenetica.ro (I.C.); bogdan.chiper@yahoo.com (B.C.); drlgheorghe@gmail.com (L.G.); 2 “Carol Davila” University of Medicine and Pharmacy, 050474 Bucharest, Romania; nicoltiuca@gmail.com (N.T.); sorinadiac@yahoo.com (S.D.); ffurtunescu@gmail.com (F.F.); cora.pop@gmail.com (C.P.); 3 University of Economic Studies, 70167 Bucharest, Romania; 4 Department of Gastroenterology, Grigore T. Popa University of Medicine and Pharmacy, 700115 Iasi, Romania; huiban.laura@yahoo.com (L.H.); lungu.christina@yahoo.com (C.M.); gilda_iri25@yahoo.com (I.G.); ancatrifan@yahoo.com (A.T.); 5 St. Spiridon Emergency Hospital, 700111 Iasi, Romania; 6 Department of Internal Medicine II and Gastroenterology, University Emergency Hospital, 050098 Bucharest, Romania; 7 Department of Gastroenterology, Research Center of Gastroenterology and Hepatology, University of Medicine and Pharmacy, 200349 Craiova, Romania; larisasandulescu@yahoo.com; 8 Department of Gastroenterology, Emergency County Hospital, 200642 Craiova, Romania; 9 Department of Gastroenterology, University of Medicine and Pharmacy, 200349 Craiova, Romania; ionirogoveanu@gmail.com; 10 Department of Cardiology, Emergency County Hospital, 200642 Craiova, Romania; 11 Department of Public Health and Management, National Institute of Public Health, 050463 Bucharest, Romania

**Keywords:** hepatitis B virus (HBV), screening, vulnerable population, prevalence

## Abstract

**Background:** Hepatitis B Virus (HBV) remains a major global health challenge, with significant morbidity and mortality associated with chronic infections. **Methods:** This study examines the epidemiology, screening, and risk factors associated with HBV in Romania, focusing on a comprehensive national screening program, LIVE(RO)2, involving 320,000 individuals (majority of them considered vulnerable population). A questionnaire was used to collect information on the potential risk factors for HBV transmission. **Results:** The overall prevalence rate of HBV chronic infection among all the participants tested was 1.67% (confidence interval: 1.63–1.72%), with significant differences (*p* = 0.0001) between participants from the main geographical regions of residence (North-East 1.89%, South 1.38%, South-East 2.06%, and South-West 1.54%). Male persons aged 30–49 or 60–69 years old, from the rural and Eastern parts of Romania and non-Romanian ethnia, with a low level of education, unvaccinated, not married, unemployed, with family members with hepatitis, with personal histories of blood or blood product transfusion, surgical interventions, tattooing, hospitalizations, imprisonment, haemodialysis, unsafe sexual contacts, or with sexual transmitted infectious diseases were risk factors associated with HBsAg seropositivity. **Conclusions:** Our findings highlight significant demographic and epidemiological patterns of reduced HBV prevalence even in vulnerable persons, as well as modified risk factors and the impact of socio-economic factors.

## 1. Introduction

The hepatitis B virus (HBV) remains a major global health challenge with significant morbidity and mortality associated with chronic infections that can lead to severe liver diseases, including cirrhosis and hepatocellular carcinoma [1]. Cirrhosis imposes a significant health burden on many countries, and this burden has increased globally since 1990, partly due to population growth and aging. Despite the availability of effective interventions for the prevention and treatment of hepatitis B and C, these diseases remain the primary causes of the cirrhosis, particularly in low- and middle-income countries [2]. The impact of hepatitis B and C is expected to diminish worldwide and be overtaken by that of metabolic associated steatotic and alcohol-related liver diseases in the near future. Cost-effective interventions are needed to continue the prevention and treatment of viral hepatitis.

The epidemiological landscape of HBV varies substantially across different geographical regions, reflecting the diverse impact of socio-economic factors, healthcare policies, and preventive measures [3]. The highest HBV prevalence rates are observed in Sub-Saharan Africa and East Asia, exceeding 8%, largely due to perinatal transmission and horizontal transmission during early childhood [4]. In contrast, countries in Western Europe and North America have significantly lower HBV prevalence rates, often below 2%, primarily due to effective vaccination programs and blood safety measures [5,6]. However, even within Europe, substantial disparities exist. Eastern European countries, including Romania, report higher HBV prevalence rates compared to their Western counterparts [7]. In Romania, the epidemiology of HBV presents unique patterns of prevalence and transmission and poses a significant public health problem, with some of the highest infection rates in the European Union [8], warranting a focused examination in order to effectively inform public health strategies. 

To address this significant public health issue, the LIVE(RO2) program has been implemented in Romania, a comprehensive national screening effort targeting HBV and HCV (hepatitis C virus) that is funded through the POCU/308/4/9/“Increasing the number of people benefiting from health programs and services oriented towards prevention, early detection (screening), diagnosis, and early treatment for major diseases” call. This project was financed by the European Social Fund (ESF), which mandates a focus on vulnerable populations defined by specific criteria set by the National Ministry of Funding. These include individuals from rural areas, those living in poverty, people with disabilities, the uninsured, the unhoused, people of Roma ethnicity, individuals without identity documents, single-parent families, individuals suffering from addiction, and victims of domestic violence and human trafficking. This viral hepatitis screening project had priority status at a national level and was aimed at enhancing patients’ access to high-quality medical services. The initiative was planned and financed through the National Strategy on Social Inclusion and Poverty Reduction chiefly because poor and vulnerable populations face higher risks of illness and early death compared to the general population [9]. Poverty also contributes to poor health through various social factors and elevates the risk of spreading hepatitis virus infections. Integrating chronic viral hepatitis into ESF initiatives supports the fund’s goals by addressing the health disparities that contribute to social exclusion, poverty, and unemployment. Efforts to combat HBV/HDV and HCV hepatitis in Romania can thus play a crucial role in achieving the ESF’s mission of fostering a more inclusive, equitable, and prosperous society.

This initiative aimed to identify individuals with HBV infection, especially among vulnerable populations, and provide them with appropriate medical care and preventive measures. The program also sought to collect data on the prevalence of HBV and associated risk factors to inform future public health strategies.

## 2. Materials and Methods

### 2.1. Study Population and Sample Design 

The seroprevalence and associated risk factors of HBV infection were investigated by a screening program that covered the Southern (POCU/755/4/9/136208) and Eastern (POCU/755/4/9/136209) parts of Romania, conducted between July 2021 and November 2023. 

The participants were from vulnerable adult group categories (≥18 years) according to the established definitions: persons from rural areas; poor people; people with disabilities; uninsured individuals; unhoused people; people of Roma ethnicity; people without identity documents; individuals from single-parent families; individuals suffering from addiction to alcohol, drugs, and other toxic substances; victims of domestic violence; and victims of human trafficking. During the aforementioned period, 320,000 patients with unique identification codes were investigated in the screening program. The patients investigated during the screening resided in one of 24 counties of the country, in the south and east, representing 58.5% of Romania’s 41 counties. It is noteworthy that Bucharest, the capital of Romania, which is located in the southern part of the country, was not included in the screening because it was considered that the population of Bucharest is not particularly vulnerable, as the capital city has much greater access to high-quality medical services compared to the counties included. The majority of the individuals included in the screening, specifically 230,310 people (71.97%), lived in the rural area in villages, while only 89,690 people (28.03%) resided in cities. 

Over 1500 healthcare professionals, including 1145 family practitioners (FP); specialists from the fields of gastroenterology, internal medicine, medical imaging, infectious diseases, and oncology; and nurses employed in public medical institutions or contracted with the Health Insurance Administration participated in the screening in the North-East, South-East, South, and South-West regions of the country. 

The healthcare establishments participating in the screening process as staging and testing grounds for patients detected as being HBV-positive through prior rapid diagnostic tests were as follows: in Bucharest, the Fundeni Clinical Institute and Bucharest University Emergency Hospital; in Craiova, the County Emergency Clinical Hospital; in Iasi, “Sf. Spiridon” County Emergency Clinical Hospital, and in Constanta, “Sf. Apostol” County Emergency Clinical Hospital. Written informed consent was obtained from each participant before their enrollment. The survey was approved by the Local Ethics Committee (No 8537/11.08.2020) and was in full conformity with the ethical guidelines of the 1975 Helsinki Declaration.

### 2.2. HBV Testing and Questionnaire 

Rapid diagnostic tests (RDT, IHB04, Turklab HBsAg, Izmir, Turkey) were performed by FP. A questionnaire was used to collect information on the sociodemographic characteristics of the participants and the potential HBV transmission risk factors. The face to-face interviews were conducted by the FP at the same time as the RDT collection. Sociodemographic information included age, sex, urban or rural address and geographical area of residence, marital status, ethnicity, educational level, and employment status. The questions on the risk factors for HBV infection included the following: the presence of individuals with chronic hepatitis B or C in the household; personal history of anti-hepatitis B vaccination; and personal history of invasive medical procedures (dental treatment, elective and emergency surgery, frequent injections at a medical facility or at home (“frequent injection” was defined as someone who had had more than six “injections”/year before the introduction of disposable needles and syringes), blood or blood product transfusions, hemodialysis, solid organ transplantation, etc.); ear and body piercing, tattooing, acupuncture, and visits to beauty salons (contaminated cutting instruments); sharing a toothbrush or razor blades with other family or community members; accidents or injuries with serious trauma; occupational hazard; a history of IVDUs (intravenous drug users); age of first sexual intercourse; risky sexual behavior (sex with prostitutes or unknown individuals, men who have sex with men, more than 10 lifetime sexual partners); time spent in prison; and alcohol abuse.

Patients detected as HBsAg positive at RDTs were sent to the aforementioned hospitals for staging (abdominal ultrasound and transient elastography) and HBV serological characterization (HBeAg, antiHBeAb, antiHBsAb, HBV DNA), as well as reflex testing for HDV (hepatitis delta virus) antibodies (ELISA kits and real-time PCR-based kit, Bosphore HBV Quantification Kit, Anatolia Geneworks, Istanbul, Turkey). Chronic HBV infection was defined as the presence of HBsAg. Antiviral therapy and linkage to care was initiated according to the National Protocols from the Health Insurance House, which was one of the objectives of the project but was not financed by the project.

### 2.3. Statistical Analysis 

The crude prevalence of chronic HBV infection was calculated for various subgroups, including sex, age groups (18–29 years, 30–39 years, 40–49 years, 50–59 years, 60–69 years, 70–79 years, 80–89 years, 90–105 years), geographical region of residence, area of residence (rural or urban), ethnic groups, educational levels, marital status, social status, and all investigated risk factors. These calculations were performed using the STATA “proportion” command, which estimates proportions along with standard errors and 95% confidence intervals for each variable level within the identified categories in the variable list. Univariate comparisons between categorical variables were carried out using the chi2-test and the Wilcoxon rank-sum test, as appropriate. The trend across ordered groups such as age groups and education level was calculated using a Wilcoxon-type test. We evaluated the association between the risk of having a chronic HBV infection and the characteristics of the participants or risk factors using an unconditional multiple logistic regression model. The dependent variable of our model was the presence or absence of markers of chronic HBV infection. The independent variables were the characteristics or risk factors to be investigated together with demographic data such as sex, age group, and area of residence (rural, urban). We tested the association for each investigated characteristic or risk factor separately. All statistical tests were two-sided, and a value of P less than 0.05 was used to indicate statistical significance. All statistical analyses were carried out using STATA/SE 11 software (StataCorp, College Station, TX, USA).

## 3. Results

### 3.1. Demographic and Epidemiological Data

The screening program covered a diverse population, with the majority residing in rural areas (71.97%) and a significant proportion being female (63.09%). The number of persons screened for HBV by geographical areas from Romania, as well as by their declared vulnerability, are shown in Table 1.

The population included in the screening was as follows: 28.33% from the North-East area, 15.42% from the South-East, 29.77% from the South, and 26.48% from the South-West area of the country. The average age of participants was 54.17 years; the average and median age for women was 54 years. For men, the average age was 54 years, and the median age was 55 years. Of the patients included in the screening (i.e., 189,819 people), 59.32% were between 40 and 69 years old, and 61.36% (i.e., 196,373 people) were over 50 years old. The average and median age in rural areas was 54 years (19–103 years), while the average age in urban areas was 54.6 years (19–101 years), with a median age of 55 years.

The ethnic composition was predominantly Romanian (96.87%), with 2.71% Roma and 0.42% from other ethnic backgrounds. Educational attainment varied, with a notable portion of the population having only primary or secondary education. Regarding the level of education, 7.48% had completed 1–4 grades; 28.10% had completed 5–8 grades; 51.7% had completed high school and post-secondary studies; and 10.3% had completed university and postgraduate studies. However, there were also 3465 people (1.08%) who had not pursued any kind of education.

### 3.2. Overall Prevalence

The prevalence of HBV antigen (HBsAg) for the 320,000 people investigated in the screening was 1.67% (1.63–1.72), with 1.62% (1.57–1.68) in rural areas and 1.79% (1.71–1.88) in urban areas, with the difference between rural and urban being statistically significant at *p* = 0.001. For female patients, the prevalence of HBV was 1.51% (1.46–1.57), significantly lower compared to males, which was 1.94% (1.86–2.02), with *p* = 0.0001. The highest HBV prevalence was found in the South-East area, at 2.06% (1.94–2.19). In the North-East area, the HBV prevalence was 1.89% (1.80–1.98). The HBV prevalences in the other investigated areas were significantly lower, namely, 1.54% (1.46–1.62) in the South-West and 1.38% (1.31–1.46) in the South. Significant HBV Ag prevalences also existed in ten counties out of the 24 where the screening was conducted, namely: Bacau with 2.15% (1.92–2.41), Botosani with 2.57% (2.27–2.92), Braila with 2.07% (1.66–2.59), Vrancea with 2.66% (2.22–3.18), Vaslui with 2.25% (1.95–2.60), Neamt with 2.42% (2.11–2.77), Gorj with 2.38% (2.10–2.69), Galati with 2.43% (2.20–2.67), Calarasi with 1.96% (1.59–2.40), and Constanta with 1.95% (1.74–2.19).

Regarding the age of the investigated patients, the highest prevalences were found in the age groups of 30–39 years with 3.15% (2.98–3.33), 40–49 years with 1.94% (1.83–2.06), and 60–69 years with 2.03% (1.93–2.15). High prevalences were also observed in relation to the level of educational attainment. People who declared that they had not attended any school had the highest HBV prevalence of 5.83% (5.10–6.66); those who attended only primary education had an HBV prevalence of 1.97% (1.80–2.16); and those who attended only middle school had an HBV prevalence of 1.75% (1.67–1.84).

The prevalence of HBsAg for those with undeclared ethnicity was 2.23% (1.56–3.17); for those of Roma ethnicity, it was 2.71% (2.38–3.07); for unmarried patients, it was 1.95% (1.82–2.10); for widowed patients, it was 2.59% (1.84–3.64); for patients who declared “other” for marital status, it was 3.64% (2.41–5.46); and for those divorced or separated, it was 1.85% (1.63–2.09).

The overall results of the prevalence of chronic HBV infection in the screened population are shown in Table 2. 

### 3.3. Risk Factors Associated with HBsAg Positivity in the Screened Population

The analysis of the administered questionnaire revealed the following risk factors associated with the presence of HBsAg: male sex; urban area; Eastern geographic area; age of the screened persons between 30–49 years and 60–69 years; ethnicity (Roma and other ethnicities different from Romanians); low level of education; marital status (widowed or unmarried); social situation (unemployed or inactive); unvaccinated for hepatitis B; previous diagnosis of hepatitis and previous treatment for hepatitis; contact within the family and/or outside the family with an infected person; sexual contact with an infected person; contact with other people’s blood as well as blood transfusions or blood derivatives; hemodialysis; previous surgical interventions (any type including dental ones); repeated hospitalizations; serious road, work, or domestic accidents requiring hospitalization; accidental needle stick or cut with an object contaminated with blood; administration of injections that were not prescribed by a doctor; being incarcerated or sentenced to prison; tattoos or piercings, including ear piercings; casual or unprotected sexual contacts with a single partner or multiple partners; and previous diagnosis of sexually transmitted infections (STIs).

Table 3 shows the risk factors according to the questions from the epidemiological questionnaire.

## 4. Discussion

Although the mortality of chronic liver disease caused by HBV and HCV has decreased in the last three decades (from 1990 to 2019), the number of deaths will continue to increase until 2030 [10]. No global burden of disease (GBD) region is projected to achieve the WHO target of a 65% reduction in mortality from chronic HBV and HCV infections by 2030. End-stage outcomes such as liver-related deaths, hepatocellular carcinoma, and decompensated cirrhosis in adults are projected to increase by 14–17%. According to WHO, in 2019, HBV infection-related causes resulted in 820,000 deaths [11]. The GBD study [2] indicated that central and eastern Europe have higher age-standardized mortality rates for HBV-related cirrhosis and liver cancer compared to western Europe. Although some countries have demonstrated effective frameworks for hepatitis prevention and control, many countries still lack adequate action plans, strategies, and funding for implementation. The current screening program results show significant demographic and epidemiological patterns of HBV prevalence in Romania, providing a comprehensive understanding of the disease’s spread across various regions and among different population groups. The present findings are compared with those of previous studies, including the single population-based study performed by Gheorghe et al. on HBV in Romania [8], to contextualize and evaluate the progress and shifts in HBV epidemiology over time. 

The present Romanian screening program covered a diverse population predominantly from rural areas (71.97%), with a significant proportion being female (63.09%). The average age of participants was 54.17 years, with a notable concentration of individuals between 40 and 69 years old. In our previous epidemiological study [8], there was also a predominance of females included, but the proportion of rural and urban origins was similar. 

This is similar to a Hawaiian study [12] that focused on foreign-born Asians and Pacific Islanders accessing services at the Kalihi-Palama Health Center, with 58.2% of participants being female and middle-aged or older adults (mean age 44.5 years). In contrast, another US study [13] screened 202,868 adults from 174 countries, with a median age of 55 years and 41.2% male participants. The US study focused on an urban safety-net system with a high proportion of foreign-born individuals, reflecting a different demographic and geographical context compared to the largely rural Romanian population. It is important to mention that the foreign-born population in the U.S. increased by 25% from 2009 to 2018, with significant proportions originating from Asia, Africa, and other high-HBV endemic regions.

Schweitzer and colleagues [14] provided comprehensive estimates for HBsAg seroprevalence over broad time periods across 161 countries, reporting a seroprevalence of 2.06% and an estimated 18.48 million people living with chronic HBV in the WHO European Region in 2010; this is lower compared to our previous double HBV prevalence reported in the population-based study. In Europe, the overall prevalence was estimated at 1.5% in a study by Tout et al. [15], similar to the present Romanian findings, but they emphasized the substantial impact of migration, with migrants from high-prevalence areas significantly increasing local HBV rates in low-endemic regions.

The present Romanian project reported an HBV prevalence of 1.67% among the 320,000 people screened, with higher rates in urban areas (1.79%) compared to rural areas (1.62%). The highest prevalence was found in the South-East region of Romania (2.06%), aligning with previous findings that identified Eastern Romania as a high-risk region. However, this is in contrast to our previous paper showing a significantly higher prevalence of HBV in the rural part of Romania (4.78% vs. 4.01%, *p* = 0.031). 

The prevalence of chronic HBV infection in Europe ranges from low (<1–2%) in Western (UK, Germany, France) and Northern Europe to intermediate (2–5%) in some parts of Eastern and Southern Europe. Countries like Italy, Spain, and Greece have slightly higher HBV prevalence rates, ranging from 1–3% [7,16,17]. The overall prevalence rate of HBsAg among 12 246 Spanish participants aged 20–74 years (58.4% females) was 0.6% (95% CI [0.4–0.7]). The current HBV burden in Spain, for example, has remained low but virtually unchanged over the past 15 years [18]. Certain population groups, such as migrants from high-prevalence countries, people who inject drugs, and men who have sex with men (MSM), have higher rates of HBV infection. In UK [19], an analysis that utilized data from 419 general practices, covering 6,975,119 individuals, was able to screen only 192,639 persons (2.8%) from the total population for HBsAg; out of these, 8065 (0.12%) were seropositive. However, screening rates varied widely, with the highest rates among close HBV contacts (38.6%), men who have sex with men (MSM) (19.0%), and women with pregnancy records (14.3%). Another study [20] was conducted in May 2019 across four districts in Italy with only 600 participants, but 61.2% of them were female, with an average age of 51 years. Despite the prevalence of the identified risk factors, only 16.3% of the participants had previously been tested for HBV or HCV. Of those tested, 24.5% were found to be positive. The Hawaiian study [12] found a much higher prevalence of chronic hepatitis B (CHB) of 10.7% among 997 participants. This stark contrast underscores the higher risk among foreign-born APIs in Hawaii compared to the general population in Romania. In contrast, the US study reported an overall HBV prevalence of 0.9%, with significant variability based on the country of origin and an average chronic hepatitis B prevalence of 3.07% among the foreign-born population in the U.S. in 2018. The study highlighted that approximately 59% of foreign-born individuals with CHB in the U.S. emigrated from Asia, 19% from the Americas, and 15% from Africa. The highest prevalence was observed among individuals from the Western Pacific region (6.6%), with particularly high rates among those from Vietnam (12.8%), China/Taiwan/Hong Kong (10.5%), and Cambodia (10.2%).

In Romania, HBV’s prevalence was higher among males than females in both our studies. Similarly, the Hawaii study [12] found that males had a higher prevalence than females, although the difference was not statistically significant. Age was a significant predictor in both studies. In Romania, the highest prevalence was in the 30–39-years age group (3.15%), whereas in Hawaii, the 35–44-years age group had the highest prevalence of chronic hepatitis B, with an adjusted prevalence odds ratio (POR) of 3.1 (95% CI: 1.1, 11.0).

The gender disparity in HBV prevalence, with males exhibiting a higher rate (1.94%) compared to females (1.51%), is consistent with our own previous findings [8], reaffirming the necessity of targeted interventions for male populations. Age-specific prevalence was found to peak at 3.15% in the 30–39-years age group and 2.03% in the 60–69-years age group in the current study, similar to the 2012 Romanian study, which identified higher rates in middle-aged adults. This persistent trend underscores the need for focused public health strategies in these age cohorts. The US study by Wong et al. [21]. also noted a gender disparity, but did not provide specific prevalence rates by gender. 

The higher prevalence rates of HBV in urban areas are consistent with trends observed in other regions, such as the United States, the European Union, and China. Several factors contribute to these urban–rural disparities in Romania: population density and mobility (a greater influx of people, some of whom may carry infections from rural areas), as well as healthcare access and behavioural factors. Paradoxically, while urban areas generally have better access to healthcare facilities, the prevalence of risky behaviors such as IVDU and unprotected sex may be higher in these settings, contributing to greater HBV transmission. The influence of gender and age on the prevalence of HBV observed in our study can be linked to behavioural, social, and biological factors, as well as specific socio-cultural dynamics. Men are more likely to engage in high-risk behaviors such as IVDU, unsafe sexual practices, and alcohol consumption, which are significant risk factors for HBV transmission; men may be also more exposed to occupational risks that involve contact with blood or bodily fluids in professions such as healthcare, manual labor, and certain types of service work. The higher prevalence of HBV among younger adults in our study could be due to greater engagement in risky behaviors and increased social and sexual activity. This age group is often at the peak of their social interactions and may have had less consistent access to vaccination and healthcare during earlier life stages.

The correlation between low educational attainment and higher HBV prevalence observed in the current study (5.83% in individuals with no formal education) supports the 2012 study’s conclusion that education level is a critical determinant of HBV risk. Both studies highlight the importance of educational interventions to raise awareness and promote preventive measures such as vaccination among less educated populations.

The US study [21] did not specifically address educational attainment, but it emphasized the need for targeted patient education and interventions based on country-specific data, suggesting similar socio-economic influences on HBV risk. The Hawaii study [12] did not specifically address educational attainment either, but did emphasize the ethnic disparities, with the majority of participants being born in Micronesia (53.3%) or South/Southeast Asia (26.2%).

The Roma population in Romania, like many other marginalized groups globally, often faces severe socio-economic disadvantages. These communities typically experience higher levels of poverty, which limits their access to basic healthcare services, including vaccinations and routine health screenings. The current study identifies a higher HBV prevalence among the Roma population (2.71%), consistent with the 2012 findings that also reported elevated rates in this ethnic group. Roma persons in Romania are considered vulnerable for several interrelated reasons, stemming from a history of marginalization, discrimination, and socio-economic challenges. This vulnerability is exacerbated by significant health disparities, particularly the high prevalence of HBV and HCV among Roma communities, as documented in recent papers by Gheorghe L. et al. [22] and Tantau A. et al. [23]. This disparity points to the need for culturally sensitive and inclusive public health policies to address the specific needs of the Roma community. Additionally, the migration of Roma individuals to Western Europe presents further challenges in managing these health issues. This mobility complicates efforts to provide consistent healthcare and maintain continuity in treatment, making it challenging to control the spread of HBV. Migratory patterns also expose Roma individuals to varying levels of healthcare services, depending on the countries or regions they move to, which can further influence infection rates. Migration-related factors have been identified as significant in other studies on infectious disease prevalence among migrant populations [7,24,25]. 

This is particularly concerning given the broader trends in HBV prevalence and related outcomes globally. Efforts to improve case identification and treatment for migrants have mostly been confined to small outreach programs in urban areas, leaving the majority of migrants with viral hepatitis unaware of their infection.

Additionally, the high prevalence among vulnerable groups, such as unemployed or inactive individuals, aligns with previous research, emphasizing the impact of socio-economic factors on HBV risk. The US study [21] also underscored the high HBV prevalence among foreign-born individuals from endemic regions, indicating that ethnicity and migration status are critical factors in HBV epidemiology. The Romanian data further emphasized vulnerabilities associated with marital status, unemployment, and previous medical procedures, which were not as extensively detailed in the US study. 

In the Spanish study by Cuadrado A et al. [18], the identified independent risk factors for HBV infection were age, nosocomial risk, and non-Spanish nationality. In another study [26], among 330 migrants evaluated, only 30% had previously been screened for HBV upon their arrival in Italy. Post-intervention, 6.9% were diagnosed as HBsAg carriers, and 61.8% were eligible for vaccination. In the UK [19], HBsAg seropositivity was highest among MSM; individuals from high-prevalence countries; close HBV contacts; those with a history of injecting drugs (IVDU); and people diagnosed with HIV, HCV, or syphilis. On the other hand, socioeconomic factors played a significant role, with higher seroprevalence in the most deprived neighborhoods and among minority ethnic groups from London.

The study from Southern Italy [26] highlighted the limited access to HBV and HCV testing, especially for individuals with multiple risk factors, such as dental treatments, unprotected sexual intercourse, and injections with glass syringes.

The analysis of risk factors in the present study reaffirms several associations identified in the old Romanian study [8], including male sex, rural residence, contact with infected individuals, and certain medical procedures (e.g., hemodialysis, blood transfusions). New risk factors, such as casual or unprotected sexual contact and previous diagnoses of sexually transmitted infections (STIs), have been highlighted, suggesting evolving behavioural patterns that influence HBV transmission similarly to in Western Europe. 

The Hawaii study [12] also identified household contact with an HBV-infected person as a significant risk factor, with an adjusted POR of 3.3 (95% CI: 1.1, 9.2). This finding aligns with the Romanian study’s emphasis on the importance of household and familial contact in HBV transmission.

The worldwide prevalence of HBV and HCV among patients with cirrhosis also varies significantly by region, with the prevalence of HBV being notably higher in African and Asian regions (8–61%) compared to Europe and the Americas (3–14%). Globally, about 42% of patients with cirrhosis are infected with HBV, which emphasizes the significant role HBV plays in liver disease progression. In many countries, particularly in regions like Africa and Asia, HBV and HCV account for more than half of cirrhosis cases [27]. The present prevalence data also reflect broader regional trends. In Central and Eastern Europe, countries like Romania have shown a higher prevalence of HBV-related cirrhosis compared to Western Europe. Moreover, alcohol consumption and metabolic-associated fatty liver disease are increasingly recognized as contributing factors to cirrhosis, particularly in regions where viral hepatitis is less prevalent. In Europe, the Americas, and Oceania, heavy alcohol use remains a major contributor to liver disease, with rates of alcohol-related cirrhosis ranging from 16% to 78% across various countries. As efforts to reduce the burden of viral hepatitis continue, addressing these additional risk factors will be crucial to effectively manage and reduce the global burden of cirrhosis.

The comparison between the current and previous studies indicates some consistent trends in HBV epidemiology, such as gender and age-related risks, educational disparities, and ethnic vulnerabilities. However, emerging patterns, such as the increasing urban-rural divide and new behavioural risk factors, signal shifting dynamics in HBV transmission. These insights are crucial for refining public health strategies, improving vaccination coverage, and implementing targeted educational campaigns to effectively reduce HBV’s prevalence in Romania. Therefore, governments and international organizations need to strengthen the effectiveness of vaccines, screening, and treatment, especially in potential emerging hotspot regions, and address migrants from countries with high prevalence rates, primarily due to the high turnover and mobility of these populations through Europe. Key barriers that should be addressed in this regard are lack of knowledge and awareness among migrants, cultural and religious misconceptions, logistical challenges, and language barriers. The association between HBV infection and poverty in Romania and in Europe in general is demonstrable.

The results of the present paper have directly informed the strategic direction of the ongoing LIVE(RO)3 project, which aims to further enhance the effectiveness and efficiency of chronic liver disease screening in Romania. The primary objective of LIVE(RO)3, entitled “Integrated Strategic Plan: Development, Monitoring, and Quality Control in the Screening of Chronic Liver Diseases”, is to implement a standardized and updated methodological framework for screening. LIVE(RO)3 not only builds upon the data and infrastructure established by its predecessor LIVE(RO)2, but also expands the scope to ensure that the screening program is implemented with the highest standards of efficacy and efficiency across all regions of Romania. This continuity ensures that the progress made in understanding and addressing chronic liver diseases (viral, alcohol-, and metabolic-associated fatty liver disease) through previous efforts is sustained and amplified in the current project.

## 5. Conclusions

Our findings highlight significant demographic and epidemiological patterns of HBV reduced prevalence, even among vulnerable populations in Romania, emphasizing the influence of modified risk factors and socio-economic conditions. These results reflect positive strides toward HBV elimination over the past 15 years. However, to sustain and enhance these gains, efficient screening programs must address the identified risk factors and leverage facilitators to optimize both screening and linkage to care for HBsAg-positive patients. Continuous and integrated screening processes, supported by clear guidelines and coordinated efforts, remain crucial for achieving long-term health benefits. A multifaceted approach tailored to the specific needs of high-risk groups, combined with innovations like point-of-care testing and digital tools, can significantly enhance screening efforts, although further validation is necessary to ensure effectiveness. As part of our ongoing commitment to HBV elimination, we will continue these efforts through the LIVE(RO)3 project, which expands the scope of screening to address chronic liver diseases across the entire country, including viral, alcohol-related, and metabolic liver diseases. Policymakers and healthcare providers should prioritize culturally sensitive, accessible, and sustainable screening initiatives to continue advancing toward HBV elimination.

## Figures and Tables

**Table 1:** A. The persons screened for HBV by geographical area from Romania. B. The persons screened for HBV by their declared vulnerability.

A		
Geographical Areas	No of Patients	%
North-East and South-Est	140,000	43.75
South and South-West	180,000	56.25
All screened persons	320,000	100
**B**		
**Screened Population**	**N**	**%**
Non-vulnerable persons	63,763	19.93
Vulnerable persons	256,237	80.07
All screened persons	320,000	100

**Table 2:** The prevalence of hepatitis B virus (HBV) infection in the general population in four main regions (Moldavia, Muntenia, Dobrogea and Banat) in Romania. Results of a screening survey of 320,000 persons.

Characteristics	Number of Tested Persons—N	HBV Prevalence, Confidence Interval—Pr(CI)[%]	*p*
North-East	90,671	1.89 (1.80–1.98)	0.0001
South	95,278	1.38 (1.31–1.46)	
South-East	49,329	2.06 (1.94–2.19)	
South-West	84,722	1.54 (1.46–1.62)	
Grouped geographical areas	N	Pr(CI)[%]	*p*
North-East and South–East of Romania	140,000	1.95 (1.88–2.02)	0.0001
South and South–West of Romania	180,000	1.45 (1.40–1.51)	
Gender	N	Pr(CI)[%]	*p*
Men	118,100	1.94 (1.86–2.02)	0.0001
Women	201,900	1.51 (1.46–1.57)	
Area of residence	N	Pr(CI)[%]	*p*
Rural area	230,310	1.62 (1.57–1.68)	0.001
Urban area	89,690	1.79 (1.71–1.88)	
Age group (years)	N	Pr(CI)[%]	*p*
18–29	27,445	0.39 (0.32–0.47)	0.0001
30–39	38,301	3.15 (2.98–3.33)	
40–49	57,881	1.94 (1.83–2.06)	
50–59	70,942	1.54 (1.45–1.63)	
60–69	60,996	2.03 (1.93–2.15)	
70–79	47,943	1.02 (0.94–1.12)	
80–89	14,941	0.58 (0.47–0.72)	
90–105	1551	0.19 (0.06–0.60)	
Ethnicity	N	Pr(CI)[%]	*p*
Romanian	309,970	1.64 (1.60–1.68)	0.0001
Roma	8684	2.71 (2.38–3.07)	
Other	1346	2.23 (1.56–3.17)	
Education (ISCED)	N	Pr(CI)[%]	*p*
No education	3465	5.83 (5.10–6.66)	0.0001
Primary education (4–5 years)	23,923	1.97 (1.80–2.16)	
Gymnasium (4 years)	89,915	1.75 (1.67–1.84)	
High school (4 years)	149,042	1.65 (1.59–1.72)	
Post-secondary school	16,314	1.32 (1.16–1.51)	
Short-term university	12,519	1.05 (0.89–1.25)	
Long-term university	20,491	1.12 (0.99–1.28)	
Master’s	3992	1.38 (1.06–1.79)	
PhD	339	1.47 (0.61–3.50)	
Marital status	N	Pr(CI)[%]	*p*
Married/Concubinage	227,449	1.68 (1.63–1.74)	0.0001
Divorced/Separated	12,732	1.85 (1.63–2.09)	
Unmarried	39,112	1.95 (1.82–2.10)	
Undeclared	38,868	1.20 (1.09–1.31)	
Widower	1234	2.59 (1.84–3.64)	
Other	605	3.64 (2.41–5.46)	
Social category	N	Pr(CI)[%]	*p*
Employed	117,881	1.57 (1.50–1.64)	0.0001
Inactive (including pupils, students, retirees)	201,345	1.73(1.67–1.79)	
Unemployed	774	2.45 (1.57–3.82)	

**Table 3:** Risk factors for HBV chronic infection.

The Investigated Patients Who Answered the Questionnaire on Risk Factors for HBV Chronic Infection = 320,000 Patients	Prevalence of HBsAg Positive			
Risk Factors	No	Yes	Not Known	*p*
Q1 The patient has been vaccinated against hepatitis B	1.70	1.04	1.96	0.0001
Q2 The patient has been diagnosed with hepatitis	1.25	28.37	4.69	0.0001
Q3 The patient diagnosed with hepatitis received medication for the reported hepatitis	1.57	16.26	6.60	0.0001
Q4 The patient had family contact with a person diagnosed with chronic hepatitis virus infection	1.44	3.41	3.01	0.0001
Q5 The patient had sexual contact with a person diagnosed with chronic hepatitis virus infection	1.52	2.78	3.09	0.0001
Q6 The patient had contact outside the family with a person diagnosed with chronic hepatitis virus infection	1.50	3.64	2.27	0.0001
Q7 The patient has or had a profession that involves contact with other people’s blood	1.66	1.41	2.98	0.0001
Q8 The patient received transfusions of blood or blood derivatives	1.61	2.45	3.61	0.0001
Q9 The patient had at least one hemodialysis in the past	1.66	1.69	3.44	0.0001
Q10 The patient had at least one surgery in the past	1.50	1.81	3.90	0.0001
Q11 The patient had at least one hospitalization in the past	1.34	1.78	2.93	0.0001
Q12 The patient had at least one dental or oral surgery	1.45	1.88	3.53	0.0001
Q13 The patient suffered serious traffic accidents at work or at home accidents that required hospitalization and medical care	1.63	2.52	3.30	0.0001
Q14 If he patient accidentally pricked or cut himself with a sharp object or other object contaminated with blood	1.59	1.79	2.56	0.0001
Q15 If the patient was administered or has administered injections that were not prescribed by a doctor	1.67	1.24	2.63	0.0001
Q16 If the patient has ever been incarcerated (sentenced to prison with execution)	1.65	3.82	3.59	0.0001
Q17 If the patient has tattoos or piercings, including ear piercings	1.72	1.59	3.69	0.0001
Q18 If the patient used intravenous drugs even once	1.66	1.68	3.69	0.0001
Q19 If the patient had casual or unprotected sexual contact with a single partner or with multiple partners	1.61	1.98	3.00	0.0001
Q20 If the patient has been diagnosed with sexually transmitted infections (STIs)	1.64	2.93	2.63	0.0001

## Data Availability

https://screening-insp.ro/activitati-derulate/comunicate-presa (accessed on 1 July 2024).
